# The Mortality of Infants

**Published:** 1840-01

**Authors:** Henry Miller


### WESTERN JOURNAL

OF

### MEDICINE AND SURGERY.

JANUARY, 1840.

### ART. 1.—*An inquiry into some of the causes of the Mortality of Infants.* By HENRY MILLER, M. D.

AMONG the many disclosures of medical statistics, which serve to enlighten us in relation to all that can affect health and longevity, none stands out more prominently than the *precariousness of life in the earlier periods of our existence.* Life, at best but brief and in all its stages environed by causes inimical to its continuance, is then peculiarly threatened with extinction. From a comparison of the most accurate obituary registers, it may be deduced that the deaths of children under a year old, constitute about a fifth of the whole number oocurring for a series of years. In Paris, for example, during the year 1818, the number of deaths was

22,421, whereof 3,942 or 17 per cent. were under the age of one year:—the deaths in Philadelphia during a period of twenty years, include rather a larger proportion of infants than the average assumed; in New York, according to the tables compiled by Dr. Lee,* embracing a period of sixteen years, twenty-five per cent., or one fourth the whole number of deaths were children under one year—the total number of deaths being 83,783, of which 21,330 were infants. The ratio of children decreases after the first year, being but 7,866 in New York, from one to two years; 6,787 only from two to five years; and it is remarkable how much greater risk to life the first than the second five years involve; thus, of the 83,783 comprised in these tables, 35,983 were children under five years, while only 2,232 were between five and ten years old.

But it is foreign to my design to enter into statistical details any farther than to establish the fact, that a proportionally larger number of children die within the first year than adults, and *an inquiry into some of the causes of this greater mortality, it is hoped, will be not without interest and practical utility.*

It is too obvious to need particular remark, that one cause of the greater mortality of children is their exposedness to certain diseases which, whether issuing from Pandora's box or not, are dispersed over the world, and may be said, almost without a figure, to guard the avenues to life. These diseases, not excepting croup and hooping-cough, often prevail epidemically, and carry off multitudes of children. Between some of them and the infantile constitution there is a particular affinity, adults being but seldom affected by them.

---

*American Journal of Medical Sciences, No. xxxvii.

Others, especially the Exanthemata, small-pox, measels, scarlet fever, &c., are not so exclusive in the selection of their subjects; but never wholly extinct, and often raging as epidemics, children are more apt to be assailed by them, because the constitution is not generally susceptible of a second attack, and but few have been ushered into manhood without having run their gantlet. But there are sources of disease to children, that are connate and do not adhere to them when grown up, which it most concerns us to investigate, and the first of these deserving attention, is a physiological peculiarity that is strongly stamped on their constitution, viz.: *Their excitability or vitality, in all the tissues and organs which it pervades, is more vivacious but less energetic than that of adults.* In confirmation of this we may refer to the manner in which some of their most important vital actions are performed. *The contractions of the heart* are much more rapid, the pulse of the infant being nearly double in frequency that of the adult, varying from 120 to 140 beats in a minute: at the same time it is evident that the pulsations are not so strong and the blood is not sent out in as bold a current in the vessels through which it circulates. Indeed, it is plain that the increased frequency is a compensation for deficient force, as the pulse of adults, who are suffering from debility, becomes frequent when it loses strength and volume. *Respiration* is carried on with greater rapidity, the inspirations numbering from 35 to 40 in the minute or nearly double those of the healthy adult; and yet, notwithstanding this activity of the respiratory process, the infant consumes less oxygen, showing that the function is less efficiently performed. *The calorific function*, as it may with propriety be called, although no special organ be allotted to it, is less energetic,—as has been proved by Dr. Edwards, who found that the young of warm-blooded animals, selected for his experiments, have a temperature less than their parents, even when best protected from external cold, and that when they are removed and kept an hour or two from the mother, in an atmosphere of 50° to 68° Fahr., their temperature falls considerably and continues falling, until in the course of three or four hours, it stops a very few degrees above that of the surrounding air. Under similar exposure adult animals sustained but a slight reduction of temperature, their power of generating heat and resisting cold existing and being exerted in full vigour. The same experimenter, also, found that the capability of supporting reductions of temperature in the young of warm-blooded animals is inversely in proportion to their power of producing heat, and that consequently of two animals of the same species, the young will suffer less and recover more perfectly from the same degree of cold,—proving the greater resiliency of their vital power, or in other words, greater vivacity of excitability, which responds more readily to the counteractives of cold when judiciously applied.

These facts abundantly establish the vital or physiological peculiarity, which we have claimed for infants, and prepare us to appreciate its morbid tendencies. The excitability of the infantile system being greater, it results that stimulants or sedatives, all the modifiers to which it can be exposed and consequently all morbid causes, must, *cæteris paribus*, produce a more considerable effect on it than on that of the adult. The susceptibility of infants to cold alone is a very fruitful source of disease, which has been particularly pointed out by the experimental author, already quoted. "If the attentions which children require in climates and seasons little favorable to the preservation of their existence," says he, "were generally understood and put in practice, it would considerably reduce one of the most powerful sources of mortality affecting that age in our climate. It is not confined to children whom the misery of their parents cannot guard from the rigour of the weather, but it operates to a great extent without being either perceived or suspected, in families enjoying affluence, and in which it is believed that the necessary cautions are taken, because cold being relative, it is difficult from our own feelings to judge of its effects on others, and because it does not always manifest itself by determinate and uniform sensations. They do not feel the cold, but they have an uneasiness or an indisposition which arises from it; their constitution becomes deteriorated by passing through the alternations of health and disease, and they sink under the action of an unknown cause."* In the management of children, a practice directly opposed to that which these observations suggest, finds favor with some, whose example, from their rank and intelligence, must exert a pernicious influence. By them it is contended that warm clothing only induces delicacy and greater sensibility to cold, diminishes the capability of resistance when unavoidably subjected to it, and that, therefore, the best fortification is exposure by times to its influence. There can be no doubt that many children fall victims to this hardening system, and that while some of iron constitution may emerge from the ordeal with increased robustness, many more have the foundation laid for disease which adheres to them for life. With all the precautions which philosophy can prescribe and maternal solicitude put in requisition, cold will claim its full proportion of mortality.

What has been said in relation to cold is equally true of excessive heat and of all morbific causes;—They more easily

*Edwards, on the Influence of Physical Agents, &c.

and seriously affect infants than adults, and it would seem from the most careful pathological researches, that infants are peculiarly obnoxious to inflammatory diseases, when even only a few days old, which may be referred to the same productive source, viz: their more lively excitability, and feebler power of resistance. This vital peculiarity may be regarded as a *general* predisposition to disease, which may remain innocuous if care be taken to guard against the application of exciting causes; but there are *special* predispositions which oftentimes bid defiance to all the vigilance and resources of art, and these may be said to consist in *the incompleteness of organic development at birth, and the necessarily progressive establishment of the circle of functions essential to the perfection of adult life.* To illustrate the extent and importance of this physiological law, and the morbid tendencies inseparable from it, will occupy the remainder of this essay. Before entering on this investigation, it is proper to advert briefly to a phenomenon observed in all the organs of the animal economy, when engaged in the healthy exercise of their functions, viz: they attract the fluids or movable organic matter towards them, and a larger amount of blood is circulated through them than in their quiescent state; in other words they become the seat of sanguineous congestion. This more profuse supply of fluids is the consequent of the increased movements of contractility, which the performance of all the vital functions requires, and the presence of the appropriate stimulus never fails to produce, at least in the healthy state. Hence, this fundamental vital law, on account of its constancy, has given rise to the axiom *uibi stimulus, ibi fluxus.* During the continuance of this physiological congestion the density and volume of the part are increased, and its frequent recurrence contributes to animal growth and vigor. Thus the muscles of locomotion are augmented in size and acquire firmness by exercise, and become atrophied by being kept in a state of immobility, as in paralysis, where nervous stimulation is intercepted, or in fractures or painful chronic affections, where their motion is impracticable or would aggravate pain. In the next place, it must be observed that this exaggeration of the vital properties of a part, if it exceed certain limits, produces disease, and that the transition from congestion to inflammation is neither abrupt nor difficult. It is on this account that the highest degree of health, when all the functions are performed with an activity that diffuses a joyous sense of well-being over the economy, sometimes portends disease. If the locomotive muscles, for example, are too severely tasked, the congestion becomes fixed, and hence the soreness and stiffness which are complained of after inordinate or prolonged exertion, and we are told that it is not unusual for the muscles of the thighs of soldiers, after forced marches, to become painful and produce a chill and fever—to inflame and suppurate as after the most violent attacks of rheumatism.* Having premised these general observations we proceed to seek for sources of infantile disease, first, *in the establishment of the respiratory function.*

The first and most urgent want which the infant experiences is atmospheric air, without which its existence cannot be prolonged beyond a few minutes: and consequently pulmonary respiration is the first of the functions, progressively established, which is called into exercise. The development of the lungs is complete at birth, and so likewise is the pulmonary artery, destined to convey to them venous blood for its conversion into arterial; but the revivification of the blood having hitherto been effected by another provision, the lungs

---

*Broussais' Physiology.

existed in a collapsed state and the main current of the blood was turned from them by temporary channels. The fetal provision being abolished, it is indispensable that pulmonary respiration be promptly established; but this may be impeded by many causes, and hence *asphyxia neonatorum* comes in for a large share of the mortality of infants. In New York, the number of deaths from this cause, or *still-born*, in sixteen years, was 4,793, being in the ratio of one to twenty of the whole number, and one to four and a half of such as died within the first year.

In pursuing the diseases which threaten the existence of infants through this function, it must be observed that even if respiration be established, the lungs are peculiarly liable to congestion, producing either simple engorgement or extravasation into their tissue. Recent researches, particularly at the Foundling Hospital of Paris, have proved the frequency of this morbid state, and that pulmonary apoplexy, as extravasation is termed, is a much more common occurrence in children than adults. Nor is it diffiuclt to account for the increased liability of new born infants to pulmonary congestion; it is, as we may fairly presume, only an excess of the normal congestion existing in all organs during the active performance of their functions, induced the more easily, if it may be so expressed, in consequence of the raw and undisciplined state of the lungs at this period;—the stimulation and expansion of the lungs invite the afflux of blood into their tissue, the vessels of which are less able to bear the sudden inundation because their contractility has not acquired strength by exercise.

Inflammation of the lungs is not an unusual sequence of these congestions, and at this period it differs from the same disease in adults in being strictly confined to the lungs, without extending to the pleura, as very generally happens at a more advanced period of life. What renders it probable that pneumonia in very young infants is purely the consequent of congestion, is the ascertained fact, that the most usual seat of the latter is that also of the former. The right lung, for example, is much more frequently congested than the left, owing to the common custom of placing children on the right side, and the posterior portion of the lungs than the anterior, from the operation of the same mechanical cause, and these, too, are the usual seats of inflammation. These pneumonias terminate in hepatization, suppuration, or softening, by which the lungs are disorganized and rendered unfit for the support of life. At a somewhat more advanced age, but still within the year, infants may be attacked by pneumonia from the same causes as adults, particularly from atmospherical vicissitudes, and Billard* believes that *shortness of breath*, *asthma*, *idiopathic coughs*, which some young persons have from their earliest recollection, are often the results of these early pneumonias. Infants are much more liable to pleurisy than is commonly supposed, and in Foundling Hospitals cases of this disease frequently occur at the most tender age, when children are exposed to the weather in the *creche* appropriated for their reception. When best provided for they are often the victims of this disease, as might have been inferred from their special susceptibility to cold, its most usual exciting cause.

The mucous membrane lining the air passages is always more or less congested in early infancy, as is clearly evinced from its redness, thickening and irritability, visible at their outlets and traceable throughout their whole extent in post

---

*Traite des Maladies des Enfans nouveau-nes et a la Mamelle.

mortem examinations. This is no more than might have been predicted from the stimulation of the first contact of air with such a sensitive surface. Here, also, in a particular manner, congestion lays the foundation for inflammatory affections, and hence the frequency of coryza, angina, and bronchitis in infants. On account of the great vascularity and sensitiveness of this membrane, or from some more latent and unexplained peculiarity, children are subject to a form of inflammatory action, which differs greatly in its character and fatal results from ordinary inflammation affecting adults, which M. Bretonneau proposes to distinguish from its congeners by the significant appellation of *diptheritis.* In this disease, to which the genuine croup belongs, the secretions poured out by the inflamed surface concrete into a membrane that contracts an intimate adhesion to it, and when the larynx or bronchia is its seat, offers such an obstruction to respiration as to cause death by suffocation. These diptherites furnish outlets to infantile life from which adults are nearly exempt; and when it is remembered that children are at least equally subject to ordinary acute inflammation of the pulmonary mucous membrane, as adults, and that this is attended with *more* copious secretion of mucus into the bronchia and *less* or *no* power of expectoration, we are prepared to form some estimate of the magnitude of this source of mortality to them.

Secondly. Let us direct our attention to the development, &c. of *the cerebro-spinal system, the brain and spinal marrow,* and inquire into the part it contributes to the catalogue of infantile maladies.

The brain of new-born children is subject to many serious congestions, and here, as in the lungs, there may be engorgement only or extravasation of blood on the surface or in the substance or venticles of the organ. This congestion is not produced by a determination of blood, consequent to the incipient exercise of the cerebral functions, but by some mechanical impediment to the free return of venous blood from the brain. It is, therefore, passive in its character, and in this respect differs from the pulmonary congestion we have been considering. If formed before respiration has taken place, it may prevent its establishment, because the want of respiration, if communicated, is not felt by the centre of perception and consequently the muscular movements necessary for the dilatation of the chest are not excited. In overpowering cerebral congestions death takes place from asphyxia, and in estimating the causes of mortality among infants, they belong to an item already considered; but they may not be immediately fatal,—the child continuing in a languid state for several days and then expiring. In such cases the fatal issue is attributable to softening of the brain, its structure being entirely broken down, and, as it were, dissolved by the fluids penetrating it. But the connexion between the brain and lungs is such that their dependence is mutual; if cerebral congestion may impair or destroy respiration, any considerable embarrassment of respiration, on the other hand, produces an accumulation of blood in the right cavities of the heart, extending to the veins and sinuses of the brain in common with the rest of the body. Hence, the veins of the neck and head are seen to swell and the countenance to become tumid and discolored when respiration is but momentarily suspended by falls, passion, &c.

These congestions may be followed by inflammation, the excess of blood itself operating as an irritant. It must be observed that inflammation at this period attacks the membranes much more frequently than the brain itself or the spinal marrow, and that convulsions are the usual consequence of such attacks. In twenty out of thirty new born children, who died of convulsions at the Paris Foundling Hospital, well marked inflammation of the spinal membranes was discovered, and in six of them the cerebral membranes were also implicated. These meningeal inflammations have a strong tendency to terminate in effusion of serum, producing dropsy of the brain, which is almost peculiar to children and too often resists every form of medical treatment. The termination in effusion is sometimes so rapid and preceded by such slight evidences of indisposition, that the real nature of the disease was not for a long time understood, and it was classed with dropsies instead of inflammations by the best nosologists. To these peculiarities it is doubtless indebted for much of its fatality: neither the parents nor physician apprehending danger sometimes until convulsions, dilatation of the pupils, &c. admonish how futile will be all efforts to stay its destructive march.

Our inspection of the infant in this aspect has as yet been confined to the bud of its existence; let us survey it for a moment when its leaves and flowers are expanding and it sends out so many tendrils to bind it to the mother's heart. Occupied at first only with satisfying the wants common to it and the zoophite, its time is past in sleeping and feeding, and it emits but feeble glimmerings of intelligence. This dormant state of the mental faculties corresponds to the *imperfect development of the brain.* The researches of Tiedemann and others have shown that prior to the ninth month, the substance of the brain is perfectly homogeneous,—the distinction of cortical and medullary matter being but faintly if at all perceived, while its consistency is absolutely that of paste. But between the ninth and twelfth month it acquires greater firmness, the cortical contrasts decidedly, by its reddish gray color, with the medullary or white substance, and it assumes the aspect and form of that of the adult. It is at this period, when the organization of the brain is going on most rapidly and its peculiar functions are most active that it is most obnoxious to disease, and this accords with the general principle already announced: the development of the organ and the assumption or more vigorous performance of its functions invite a determination of blood to it, and this predisposes to disease in the manner already explained. Hence, children are more liable to inflammation of the brain or its membranes at this period and more exposed to convulsions arising from irritation in other organs. Until now, indeed, other organs influence the brain but little and, for want of a common head during its minority, affect an independence which they are compelled to surrender when its empire is established. It is on account of this imbecility of the brain that there is so little sympathy between the different organs in early infancy, that inflammation may take place in them, ending in suppuration and the most profound lesions of structure, without its being announced by febrile reaction. But when the brain receives its development and becomes the centre of perception, to which impressions are conveyed from all parts, to be reflected thence to all, it forges the chain of sympathy which unitizes them and makes each responsive to the impressions, whether salutary or noxious, made on the rest. And now observe the difference; irritation cannot be set up in any of the tissues, much less can inflammation take place, without arousing the heart and producing symptomatic fever, or, if its force be directed to the brain, spasms or convulsions ensue. In a word, it is only at this period that the nervous system participates fully in the peculiar excitability that characterizes all the tissues in infancy.

Third and lastly, we shall look to *the digestive organs as a source of infantile diseases.* Here it must be observed, in the first place, that digestion is performed with much greater activity in infants than in adults, as is evinced by the more frequent calls of appetite and the more rapid nutrition of the body. We should be led to expect, therefore, conformably to the principle already established and more than once alluded to, that their digestive organs would be more frequently diseased; and this is what observation has proved, it being a well known fact that more children die from diseases of these organs than any other. In New York, from the statistical tables already referred to, it appears that 6085 of the deaths were from *bowel complaints,* including under this head diarrhœa, dysentery, and cholera infantum. The digestive organs are in a state of extraordinary development at birth, the alimentary canal having received its full proportional dimensions, with its valvulæ conniventes for enlarging its surface, and the liver being much larger proportionally than in the adult, and they are the seat of an active circulation of blood. The mucous membrane is always redder, more vascular and irritable than in the adult; and hence none but the blandest nourishment can be tolerated. This congestion lays the foundation for disease, which sometimes proceeds to build without any other materials, but much oftener with the assistance of such as are furnished by the officiousness of nurses, who, not content with nature's beneficent provision, ply their charges with indigestible or too stimulating aliment or drench them with irritating drugs. From indiscretions of this kind, and sometimes notwithstanding all the precautions that can be taken, the digestive mucous membrane in very young infants is affected with inflammation, which may be confined to the mouth, stomach, or intestines, but is prone to spread from one section to another. Fatal gastritis and enteritis are not uncommon at this period, but their complication, gastro-enteritis, we are assured by Billard, is much more frequent. The morbid secretions of the inflamed membrane very often concrete into thin pellicles, covering the diseased surface, and this is not confined to the mouth, where it is so commonly observed, but extends to the esophagus, stomach, and intestines. Diarrhœa and cholera infantum are most apt to appear at a more advanced period, and M. Billard* thinks that a new development, which takes place about the same time, has something to do with these complaints. At about the fourth month, numerous mucous follicles make their appearance, throughout the whole tract of the alimentary canal, but in greatest profusion in the intestines. If existing antecedently they are so small as to escape notice; but their development is very active at this time, and in consequence of their increased vital energy, their secretions are greatly augmented. As these mucous follicles are possessed in greatest number by dogs and other carnivorous animals, whose digestion is very powerful, M. Billard is of opinion that they are designed to strengthen the digestive forces and that their development is preparatory to the change of diet which the child must shortly undergo. Irritation of these mucous follicles, or simple increased secretory action, according to him, may give rise to diarrhœa, especially the serous diarrhœa of authors, as he has verified by post mortem examination.

Synchronous with this, another development, pertaining to the digestive organs, makes its appearance; *dentition* commences and is progressively carried on until at about three

---

*Op. Cit.

years old, the child obtains its complement of deciduous teeth With regard to the morbid influences of this development there is, unfortunately, great discrepancy of opinion among pathologists. It may be observed, in general, that English writers attach great importance to dentition as a cause of disease, while the French depreciate or altogether repudiate it. Thus, of the former an eminent author, Mr. Bell, declares that "to enter upon a history of the consequences which result from the irritation of teething would be to treat generally of *all* the diseases of infancy; for there is scarcely a disease to which this period of life is subject, and scarcely a symptom appertaining to those diseases, which is not at times produced, or at least augmented by this cause."* On the other hand, a distinguished champion of the latter, M. Guersent, commences an article on this subject with this reflection: "the world ascribes most of the diseases of infancy to dentition; the difficulty of observing the diseases of early life, and the little positive information which we possess on this part of pathology, have contributed to radicate this opinion; and this prejudice resulting from our ignorance, like all other prejudices in medicine, has become popular."†

"Who shall decide when doctors disagree?" Why, doctors, of course, and, therefore we may be permitted to assume the umpirage.

Derangements of the digestive organs, especially diarrhœa and cholera infantum, a febrile state, and convulsive affections are the principal disorders ascribed to dentition, although Mr. Bell's list is much more extended. Now, it must be conceded that these diseases are much more apt to attack children during dentition than antecedently or subsequently: but this

*Anatomy, Physiology, and Diseases of the Teeth.
†Dictionnaire de Medicine.

may be only an accidental coincidence and such, indeed, is the explanation offered by M. Billard, who is strongly tinctured with the peculiar views of his French brethren in this respect. Diarrhœa, for example, he ascribes to the development of mucous follicles in the intestines, and convulsions to the activity of the organic process which is going on in the brain and spinal marrow at this period. With regard to the first, it may be observed that the congestion and increased excitability accompanying the follicular development certainly do strongly predispose to bowel affections; but it is doubtful whether disease would often result without the application of an exciting cause; if this be withheld, the predisposition will remain dormant in most instances, though it may sometimes be converted into disease by its very excess. The necessity of an exciting cause is argued from the fact that infantile diarrhœa may generally be traced to indigestible aliment, to cold, or some other cause which experience has proved to be adequate to the effect. The same strictures will apply to the second; while it is admitted that the congestion attendant on cerebral development may pass spontaneously into disease, much more frequently an exciting cause is necessary, and this may be found in local irritations of any of the organs, with which the brain sympathises: and among these gastric and intestinal irritation is known to be a frequent cause of convulsions in children. Now, the question is, can dental irritation be sufficiently great to prove an exciting cause of the diseases imputed to it? It is evident that when the process of dentition is regularly and healthily performed, it cannot be a source of disease, because being a natural development no more excitement should attend than what is necessary for its accomplishment. Some irritation of the gums and increased salivary secretion may be expected as a consequence of the accompanying congestion. But because dentition is a natural function, does it follow, as M. Billard would insinuate, that it ought *always* to be effected without accident or risk to the child? What becomes, then, of his explanation of the frequency of bowel affections? Is not the development of follicles as natural, and should it not, therefore, be as harmless as the development of teeth? Regarding the subject, then, apart from all prejudice or sectarian bias, we are constrained to admit that both may become morbid, and consequently sources of disease to infants. But in the case of dentition, if we mistake not, besides the morbid liabilities accompanying it in common with all analogous processes, there is a peculiar source of irritation which is not found in the other developments to which we have referred. A glance at its physiology will disclose this specialty and enable us to appreciate its importance. The organic arrangement for the formation of the teeth is somewhat complicated, consisting of a vascular and nervous pulp, covered by a very delicate membrane, and these again invested by a fibrous membrane of very firm texture, composed of two distinct layers. The bony part of the tooth is secreted by the fine membrane of the pulp, and as the ossification advances from within outwards, the cavity occupied by the pulp is contracted, until reduced to the dimensions of the hollow of the perfect tooth. The ossification being completed, the inner surface of the fibrous coat takes on the office of secretion and furnishes the tooth with its covering of enamel; this being done, the fibrous membrane is no longer of any use, and must be removed to make way for the tooth before it can advance above the gums. Its removal is effected by the action of the absorbent vessels.

From this succinct exposition it is apparent that growth and decay are going on at the same time in this process, and that in order to its harmony there must be perfect coincidence in the work of composition and decomposition: for, should the fibrous membrane *not* be removed in time, a barrier will be opposed to the emancipation of the teeth, and pressure continuing to be made on it by the advancing teeth, irritation first and then inflammation must be excited in its tissue. This is what happens in fact when dentition is morbid; and the inflammation extending to the gums, they become red, tumid, and painful, ending sometimes in ulceration. When it is remembered that inflammation exalts the sensibility of all the tissues, some idea may be formed of the pain that must be produced in the inflamed fibrous membrane, whose tension is at the same time increased, and this pain, Mr. Bell alleges, is exasperated by counter-pressure on the sensitive pulp itself.

The question now recurs, is there here a sufficient focus of irritation to produce any of the morbid effects ascribed to dentition? With regard to convulsions, there can be no hesitation in answering the question in the affirmative, for we are familiar with them as the consequence of other local irritations. Intense pain in any part may modify the brain so as to give rise to convulsions, nor can the minutest post mortem scrutiny always detect inflammation in this organ or its membranes in such cases. Ought it to be esteemed strange that a febrile state of system may arise from dental irritation? With what are we more conversant than symptomatic fever from local injuries attended with less pain? Ought we to be surprised that derangements of the digestive organs should proceed from this cause? The membrane that lines them is implicated in the mouth and the irritation of this point spreads with peculiar facility in infants, to say nothing of their sympathetic participation in the derangement of the nervous and circulating systems.

But, as it may be said that this reasoning is analogical only, and affords at most but presumptive proof of the point at issue, we shall adduce evidence which, we think, must be regarded as conclusive. That the morbid effects in question are sometimes produced by dentition is to be inferred: first, from the fact that they occur simultaneously with the inflammation of the gums and disappear when this subsides, to be renewed when the inflammation is again excited by the irritation of other teeth. Second, from the amelioration or complete removal of all the symptoms when the dental irritation is relieved by a free incision through the gums and fibrous membranes, an operation which, notwithstanding all the clamor that has been raised against it, is attended with little or no pain, involves no risk, and greatly facilitates the protrusion of the teeth. There is a vulgar prejudice against this operation which supposes that as the scar which follows the incision is *harder* than the gums, it will be more difficult for the teeth to make their way through it, and this prejudice is fostered by those who are incredulous as to dentition's being a cause of disease, and taken advantage of by charlatans, who have some nostrum to recommend instead of the lancet. It is not surprising that the latter should hold this up as a scarecrow to frighten parents to the use of ridiculous "soothing syrups," but it is both surprising and reprehensible that an enlightened physiologist, such as M. Billard, should countenance such an absurdity. We had supposed that it was known to every tyro in medicine, that all adventitious tissues designed to repair breaches of original structure, are possessed of *less* vitality, and that, though their *hardness* may be greater, they are *less* capable of resisting a vital force. Hence, when inflammation attacks a part or pressure excites the absorbents to action, cicatrices, if any exist, are the first to yield to ulceration or absorption, The teeth will, therefore, make their way through this *hard* scar much more easily than through the original gums.

From our subject, imperfectly as it has been handled, it would appear that disease is inseparable from the laws of organization, ordained for the production and growth of living beings, whose existence depends on the establishment of certain functions, the exercise of which may induce such derangement as to extinguish life, before the machinery it animates is put in harmonious motion. It is melancholy to reflect on the wrecks that are so thickly strewed on the broad and impetuous river of life in the very commencement of our voyage. Of the millions of frail barks launched on its treacherous bosom, with their gay pennants fluttering in the breeze, how few reach the haven of manhood, how many are ingulphed in infancy!

Although such is the constitution of nature, we may reasonably hope to mitigate the calamities of our lot, by the study and observance of her laws, and without a knowledge of those pertaining to organization, the diseases of infancy cannot be understood or scientifically treated. It must be allowed, indeed, that in spite of all the lights of science a degree of obscurity veils the diseases of this early period, from which those of a more advanced age are exempt. The diagnosis of the latter, besides being more strongly marked perhaps, is greatly assisted by the disclosures of the patient himself, an advantage which is denied to the former. But this obscurity is not as great as is commonly alleged: for, though infants have not the faculty of speech, their physiognomy, including not merely expression of countenance but the manner in which the different functions are performed, is very significant and reveals oftentimes not only the seat but nature of the diseases to which they are a prey. This inarticulate language cannot be learned from lexicons nor can it be taught, except in a very general manner, in the prelections of the schools: it must be acquired by the diligent observation of nature, for which the major number of practitioners have so little relish that they turn from it with a feeling of irksomeness, if not with disgust. Spread before them a list of diseases which have been dissected by catachetical inquisition and methodically arranged according to their several groups of symptoms, leaving them only to inquire *by responses* whether the patient is suffering from this or that disease, and the task imposed has nothing in it revolting; the whole nosological nomenclature is patiently ransacked, and the disease singled out and prescribed for; but ask them *to observe* for themselves and in default of oral language to catch the lights and shades of disease, which no language can depict, and they are ready to sing a requiem to such aspirings, composed of lamentations over the darkness of pathology, which, it may be, exists only in their own minds. This blameable apathy has, in a measure, consigned the diseases of infants to the ignorance of nurses and the tender mercies of quacks, whose congenial element is *darkness*, real or fictitious. It is incumbent on Medicine to rescue infants from such incompetent hands, and by the profound study of the peculiarities of their organization and diseases to curtail the fearful mortality to which they are exposed.

